# Effect of Metallic Coatings on the Wear Performance and Mechanism of 30CrMnSiNi2A Steel

**DOI:** 10.3390/ma16186191

**Published:** 2023-09-13

**Authors:** Huicheng Zu, Zhiqiang He, Bin He, Zhuoquan Tang, Xiuyang Fang, Zhenbing Cai, Zhongqing Cao, Luling An

**Affiliations:** 1 College of Mechanical and Electrical Engineering, Nanjing University of Aeronautics and Astronautics, Nanjing 210016, China; huicheng@nuaa.edu.cn; 2 State-Owned Wuhu Machinery Factory, Wuhu 340200, China; 3 Tribology Research Institute, Southwest Jiaotong University, Chengdu 610031, China; hzqq@my.swjtu.edu.cn (Z.H.); 15629643560@163.com (B.H.); tangzq@my.swjtu.edu.cn (Z.T.); fangxiuyang@home.swjtu.edu.cn (X.F.); caizb@swjtu.cn (Z.C.); zqcao@swjtu.edu.cn (Z.C.)

**Keywords:** 30CrMnSiNi2A, coating, Cr, wear resistance

## Abstract

The finger lock structure of aircraft landing gear is prone to wear and failure during repeated locking and unlocking processes, which is disastrous for the service safety of the aircraft. At present, the commonly used material for finger locks in the industry is 30CrMnSiNi2A, which has a short wear life and high maintenance costs. It is crucial to develop effective methods to improve the wear resistance of 30CrMnSiNi2A finger locks. This work explores the wear resistance and wear mechanisms of different metallic coatings such as chromium, nickel, and cadmium–titanium on the surface of a 30CrMnSiNi2A substrate. The effects of load and wear time on the wear behavior are also discussed. The results indicated that the wear resistance of the chromium coating was the maximum. When the load was 80 N and 120 N, the wear mechanisms were mainly oxidation and adhesive. For greater loads, the wear mechanism of the coating after failure was mainly abrasive and oxidation, and the wear was extremely severe. When the load was 80 N, for a greater loading time, the wear mechanisms were mainly oxidation and adhesive.

## 1. Introduction

Aircraft are a reliable, economical, and fast means for carrying people and goods, thus playing an important role in transportation [1,2,3]. As a result, the analysis of their structural parts and the related failure is pertinent as the system plays an important role as a bridge between the aircraft fuselage and the wheel brake system. The design performance of this system directly affects the safety of aircraft takeoff and landing. To reduce drag during flight, the aircraft needs to retract the landing gear into the fuselage or wings and open it during landing to reduce the impact force between the aircraft and the ground. This is crucial for reducing the force on the aircraft. The locking mechanism in the landing gear is pertinent for these processes. Some examples of locking mechanisms are snap ring locks, steel ball locks, and finger locks [4]. Hu et al. [5] conducted a simulation analysis on common faults of aircraft landing gear retraction mechanisms and systems. The study also examined hydraulic pump leakage, actuator leakage, and oil filter blockage. However, there is a lack of research on the structures of locking components.

The finger lock is the most commonly used locking device. It generates strong sliding friction with the outer cylinder wall during the locking and unlocking processes. The material loss caused by friction changes its structural dimensions, thus affecting the safety of takeoff and landing. Therefore, it is critical to study the wear resistance of its materials.

Han et al. [6] investigated the friction phenomenon in the interference fit assemblies with the help of a numerical analysis. After calculating the equivalent stress and contact stress after interference fit, it was found that the wear range increased for larger interference values. Hou et al. [1] studied the effects of six parameters—lock material, lock length, lock diameter, number of petals, and fingertip angle—on the unlocking force after 500 unlocks. However, the influence of the surface treatment of the base material on the axial force requires further investigation. Moreover, the work barely analyzes the differences in the wear resistance of different materials from the perspective of friction and wear data.

The material 30CrMnSiNi2A is a low-alloy high-strength steel commonly used for finger locks. In order to improve its surface quality and wear resistance, the surface treatment of the material has been carried out using metallic coatings as they are a suitable method for such performance improvement. Coatings have advantages such as low cost, easy maintenance, and excellent performance and are widely used in the aviation industry [7,8,9,10]. Common coatings include chrome plating, nickel plating, cadmium–titanium plating, and copper plating. The chromium plating layer has a large hardness, which has a positive effect on its wear resistance, but the current efficiency of the chromium plating process is very low and generates a large amount of hydrogen, which can cause hydrogen embrittlement. The nickel-plated layer is commonly used in aircraft stainless steel brazing pre-treatment to improve the brazing performance but also has better wear resistance and corrosion resistance, but the nickel-plated surface often displays a large number of defects, holes, cracks, etc., affecting its mechanical properties. Cadmium-plated titanium can be used for the protection of high-strength parts of the aircraft, and the coating has good corrosion resistance, but the cadmium-plated titanium layer has low hardness, which may have a greater impact on its wear resistance. Copper plating is often used to resist oxidative corrosion, but copper is not abrasion resistant, is easily discolored, and is not suitable for structures where wear and tear occurs over time, and the copper plating process has a poorer bond [11,12,13,14,15,16,17].

The current literature on the 30CrMnSiN2A material focuses mainly on fatigue life and mechanical properties [18,19,20,21,22,23], with barely any attention paid to the wear resistance. Xue et al. [24] investigated the wear resistance of 30CrMnSiNi2A and analyzed the wear mechanisms under different loads and friction speeds. However, this analysis of wear resistance was primarily qualitative as it lacked the calculation of wear evaluation criteria such as wear volume and wear rate. Zhao et al. [7] studied the friction and wear properties of a chromium coating on the inner cylinder of the landing gear. The wear resistance of the specimen with the Cr coating increased considerably, with 7.6 times less wear compared with that of the substrate. However, the wear mechanism and the discussion of the preparation process of the Cr coating required a deeper explanation. The work only compared the behavior of the chromium-coated specimen with that of the bare substrate, without comparing it with those of other coatings. Alexey Vereschaka et al. [25] analyzed the tool life of cemented carbide tools coated with Cr, Mo–(Cr, Mo), N–(Cr, Mo, Al), and N coatings and found that all the coatings increased the life of the tools. Duan et al. [26] investigated the effect of the addition of Ni to 30CrMnSiNi2A powder to improve the hardenability of the alloy in the manufacture of complex parts using hot pressing techniques. The experimental results showed that the tensile strength increased from 1312 MPa to 1466 MPa and 1747 MPa with an increase in the Ni content, indicating its positive effect on the mechanical properties of alloy steel. However, the use of Ni as a coating for this material for improving the mechanical properties of the material was not explored in this work. Zhao et al. [27] investigated the corrosion resistance of the 30CrMnSiNi2A surface after cadmium–titanium coating. The experimental results showed that its corrosion resistance under different environments increased due to the coating, but the wear resistance of the material was also pertinent. The work lacked a discussion of its impact on other mechanical properties of the material.

The present work focuses on the wear resistance of the 30CrMnSiNi2A substrate and the effects of three metallic coatings on it. The discussion includes the advantages and disadvantages of the coatings, especially their wear behavior, and the differences in wear resistance are derived from a quantitative analysis of the amount of wear, the rate of wear, and the selection of the coating with the maximum wear resistance.

## 2. Materials and Methods

### 2.1. Materials

The substrate used in this work was 30CrMnSiNi2A, a low-alloy ultra-high-strength steel used for the finger lock of the aircraft actuator cylinder. Its chemical composition was as follows: C, 0.3%; Mn, 1%; Si, 1%; Cr, 1.2%; and Ni, 1.5%, and the balance was Fe [22]. To investigate their effect on the wear resistance of the substrate, three coatings of chromium, cadmium–titanium, and nickel on the 30CrMnSiNi2A substrate were explored.

### 2.2. Pre-Cleaning and Coating

Before coating with Ni and Cr, the substrate was degreased for 10 to 30 min in a solution composed of 40 g/L NaOH (Huasheng, Tianjin, China), 25 g/L Na_2_CO_3_ (Huasheng, Tianjin, China), and 25 g/L Na_3_PO_4_ (Huasheng, Tianjin, China). Then it was immersed in a solution of 100 g/L CrO_3_ (Huasheng, Tianjin, China) and 25 g/L H_2_SO_4_ (Huasheng, Tianjin, China) (ρ = 1.84 g/cm^3^) for 10 s for the ash removal. Finally, it was subjected to acid pickling in 80 g/L H_2_SO_4_ (ρ = 1.84 g/cm^3^) for 2 min.

Prior to the cadmium–titanium coating, the substrate was cleaned with an organic solvent, blasted with a quartz sand abrasive to create a surface profile, and then cleaned under cold running water. Then, it was subjected to acid pickling in 40 mL/L HCl (Huasheng, Tianjin, China) (ρ = 1.19 g/cm^3^) for 10 s, ensuring that the pickling solution was not contaminated by oil and dirt. Finally, the Cd/Ti coating was carried out immediately.

The plating process mainly involved placing the specimen as a cathode into a configured plating solution; at the other end, the corresponding material was used as the anode so that the plated part was in parallel with the anode material. An electrochemical process was initiated in which crystals were precipitated on the surface of a plated part after connecting an energized wire. The coating parameters for each coating type are shown in Table 1.

**Figure 1:**
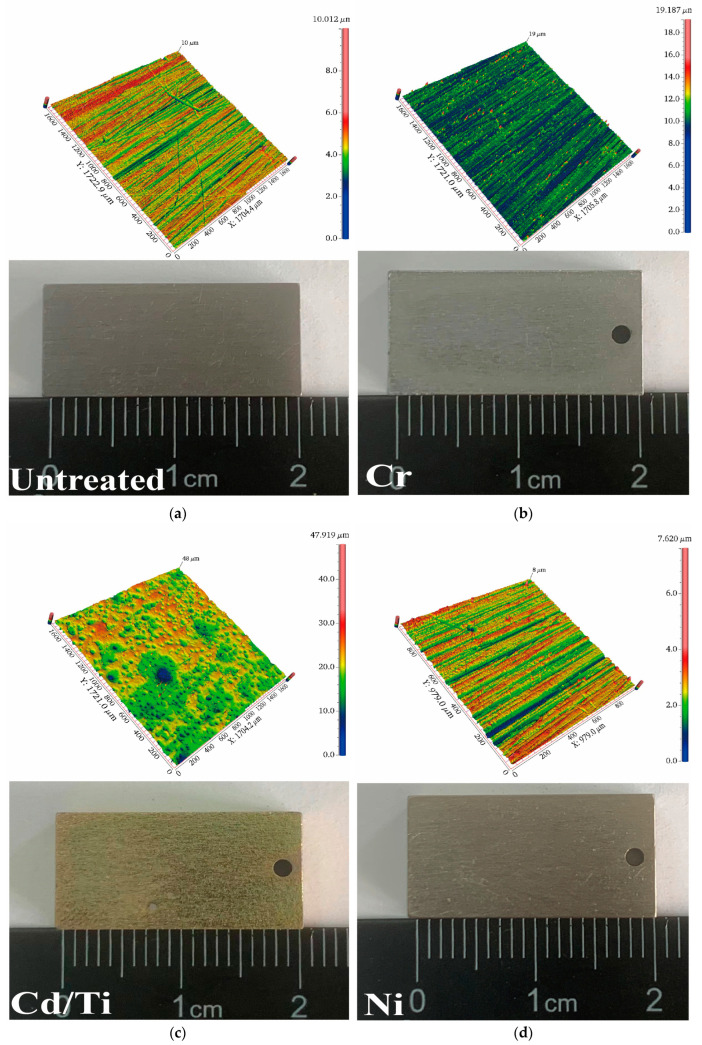
Wear specimens and the pre-test surface morphology. (**a**) Surface morphology of the substrate; (**b**) Surface morphology of chromium-plated specimens; (**c**) Surface morphology of cadmium-titanium plated specimens; (**d**) Surface morphology of nickel-plated speci.

For the wear test, the samples were cut into dimensions of 20 mm × 10 mm × 5 mm, and their surface morphology was analyzed prior to testing, as shown in Figure 1.

### 2.3. Pre-Wear Characterization Analysis

All of the following characterization analyses were performed at ambient temperature, pressure, and humidity conditions. The phase composition of the coatings was analyzed by using an X-ray diffractometer (XRD, BRUKER D8 Advance Karlsruhe, Germany), with a 2θ scanning range of 10°–90°. The surface morphology and roughness were measured using an optical 3D surface profiler (SuperView W1, Shenzhen Zhongtu Instrument Co., Shenzhen, China). A scanning electron microscope (SEM, JSM 7800F, JEOL, Tokyo, Japan) was used to characterize and analyze the surface and cross-section of the samples. The SEM wavelength scattering was at an accelerating voltage of 15 Kv. The SEM used a tungsten filament. The surface hardness experiments were conducted according to the standard of GB/T 4340.2-2012 [28]. The surface hardnesses were measured with a Vickers hardness tester (Akashi MVK-H21, Tōkyō, Japan) at a pressure of 200 g. The average of the three hardness measurements was calculated with the standard deviation as the error.

### 2.4. Wear Test

**Figure 2:**
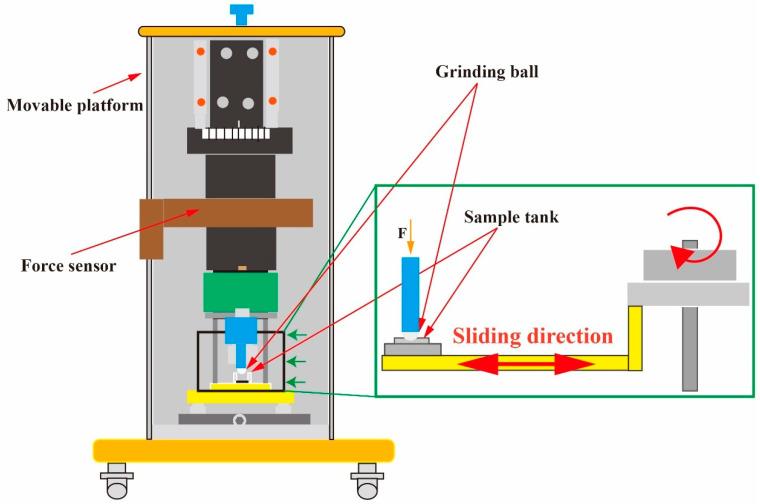
Friction and wear test bench and schematic of experimental setup.

All wear experiments were conducted on the multi-functional wear test equipment (CFT-I, Lanzhou Zhongke Kaihua Technology Development Co. China). The schematic of the equipment is shown in Figure 2. This mechanism converted rotary motion into reciprocating motion in the partially enlarged view. The direction of reciprocation is indicated by the arrows in Figure 2 using a ball-surface counter-grinding sub. The effects of three variables—coating type, load, and wear time—on the wear behavior were investigated. The specific experimental variables are shown in Table 2. Formula (1) was used to calculate the wear rate and evaluate the wear resistance of the material.

(1) $$I=\frac{V}{F\times S}\;$$

where *V* is the wear volume, *F* is the normal load, and *S* is the total distance.

### 2.5. Post-Wear Characterization Analysis

The conditions of the use of the characterization methods used in this section are consistent with Section 2.3. After the wear test, ultra-deep field optical microscopy, SEM, and Energy Dispersive Spectrometer (EDS) were used to analyze the contours and elemental compositions of the wear scars. The optical three dimensional (3D) surface profiler was employed to measure the contours of the wear scars and calculate the wear data.

## 3. Results and Discussion

### 3.1. Characterization of the Coatings

**Figure 3:**
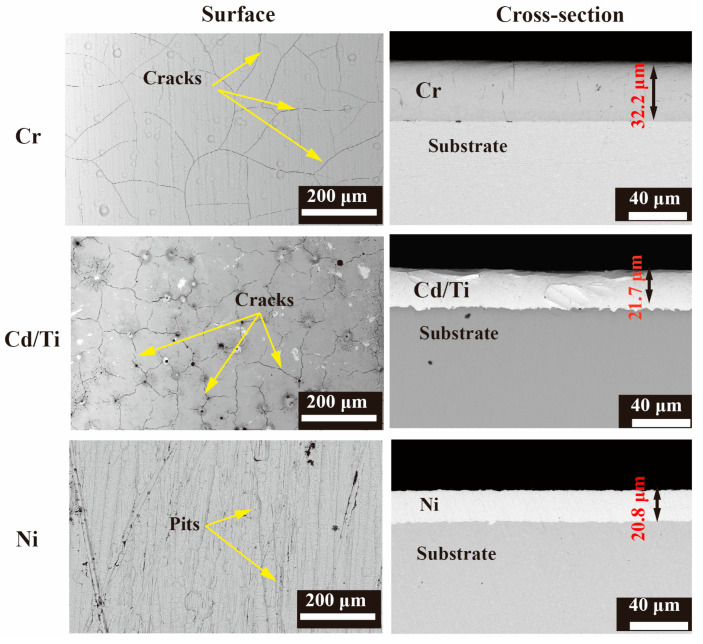
Surface and cross-section morphologies of three coatings.

After the wear test, the cross-section and surface morphology of the three coatings were characterized and analyzed using SEM, as shown in Figure 3. The thickness of the Cr coating was 33.6 μm, while that of Cd/Ti was 21.7 μm. The thickness of the Ni coating was 20.8 μm. The cross-sectional contours of the three types of coating samples were clear, and the coating/substrate interfaces for the Cr and Ni plating were uniform. Meanwhile, at the coating/substrate interface of the Cd/Ti coating, there was a distinct boundary layer.

As shown in the surface morphology on the right side of Figure 3, there were many slender cracks on the surface of the Cr and Cd/Ti samples. The probable reason is the hydrogen evolution observed during the coating process [29] and the greater thickness of the coating. When the Cr coating reaches a certain thickness, the internal stress generated by the hydrogen evolution reaction may cause microcracks to appear on its surface [30]. The surface cracks of the Cd/Ti coating were denser and more obvious, with more voids, which Was related to the Ti content. The higher the Ti content, the more compact was the coating surface, with fewer pores and cracks [31]. Meanwhile, the surface of the Ni coating was relatively smooth, but there were some pits, probably because of the defects on the surface of the substrate [32].

**Figure 4:**
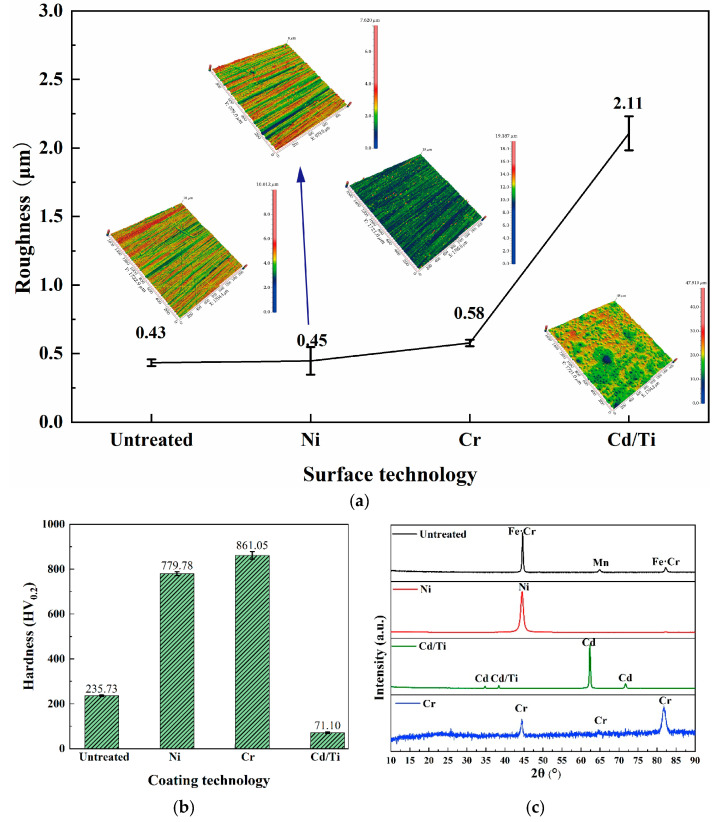
Hardness and phase composition of the surface. (**a**) Surface roughness of each specimen; (**b**) Surface hardness of each specimen; (**c**) Surface XRD of each specimen.

Further in-depth analysis was conducted on the surface properties of different coatings. As shown in Figure 4a, the surfaces of the untreated substrate and the Ni and Cr coatings were relatively smooth, with a surface roughness of about 0.5 μm. The surface of Cd/Ti was the roughest, caused by the numerous pores in the coating, which greatly reduced its wear resistance.

The surface phase analysis in Figure 4c shows that Fe, Cr, and Mn were distributed on the untreated surface. The Cd/Ti coating was composed of Cd, containing a small amount of Ti, while the Ni was relatively pure nickel. The Cr coating had a rich distribution of Cr phases.

Figure 4b shows that the hardness of the samples increased by nearly three times after the Cr coating and the Ni coating, with the Cr one being the maximum. The Cd/Ti coating showed a 50% reduction in the surface hardness. The formula for the relationship between the hardness and the wear resistance, according to the Archard wear model [33], is shown in Equation (2).

(2) $$V=\alpha \cdot \frac{N\times S}{H}$$

where *α* is the shape factor, *N* is the normal load, *S* is the contact area, and *H* is the hardness.

This formula indicates that the greater the hardness, the better its wear resistance. As shown in Figure 4b, the hardness of the Ni coating was more than the untreated surface. It was the maximum for the Cr coating, pointing to a probable highest wear resistance. Meanwhile, it was reduced for the Cd/Ti coating, indicating a lower wear resistance. This was corroborated by the higher roughness due to the pores in the Cd/Ti coating.

### 3.2. Analysis of Wear Properties

**Figure 5:**
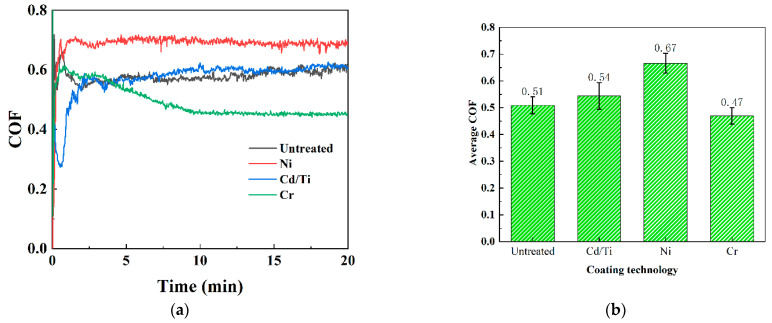
Variation of friction coefficient. (**a**) Coefficient of friction of different coatings; (**b**) Average friction coefficient of different coatings in stabilization phase.

The coefficient of friction (COF) is an important indicator for evaluating the wear performance. The friction coefficient curve obtained from the friction and wear experiments was used to calculate the friction coefficient data. As shown in Figure 5a, the running-in period of all samples can be divided into three stages: (1) the rapid upward stage, (2) the downward stage, and (3) the stable stage. However, during the initial stage of wear, the friction coefficient of the Cd/Ti coating decreased, followed by a rapid and sharp increase. The average friction coefficient during the stable stage is shown in Figure 5b, where the Ni coating had the highest value of 0.66. When the hardness and roughness were not significantly different, the average friction coefficient of the stable stage of the Cr coating was 0.47, which was less by nearly 0.2 than that of the Ni coating. The friction coefficient of the Cd/Ti coating was not significantly different from that of the untreated sample. From the friction coefficient, the Cr coating had the maximum wear resistance.

**Figure 6:**
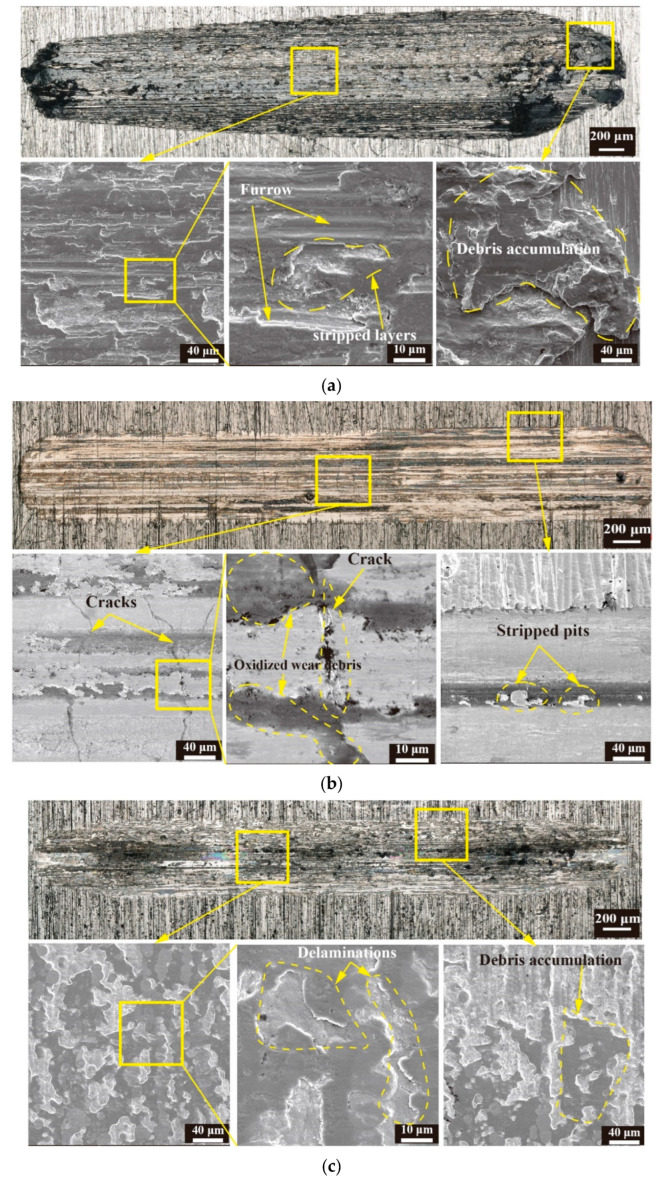

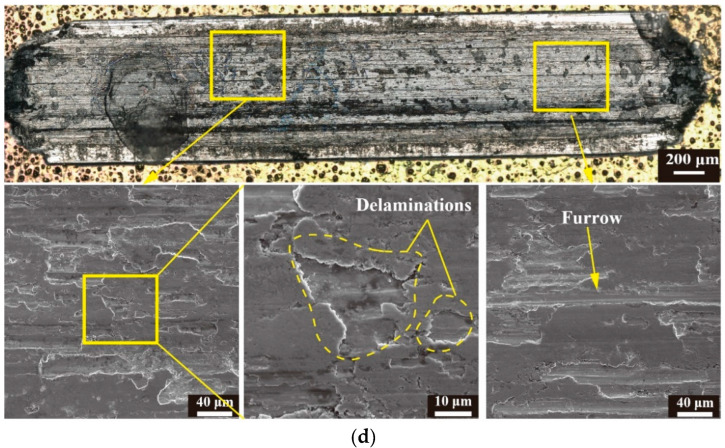
Wear morphology. (**a**) Morphology of wear scar on untreated specimens; (**b**) Morphology of wear scar on chromium-plated specimens; (**c**) Morphology of wear scar on nickel-plated specimens; (**d**) Morphology of wear scar on cadmium- titanium plated specimens.

Figure 6 shows the overall wear morphology and local enlarged image of the coated and untreated samples under wear conditions of 40 N for 20 min. Numerous black spots were visible on the wear scars of the untreated samples, as a result of debris accumulation and oxidation wear. The local enlarged image shows several peeling pits and furrows at the wear scars, with some layering. The debris accumulation in the wear scars was the most severe at both ends. The untreated samples were mainly characterized by oxidation and abrasive wear. The wear scars on the surface of the Ni and Cr coatings were significantly narrow, while the width of the wear scars on the Cr coating was the smallest. The magnified view shows that the wear scars on the Ni coating had longer cracks, along with the presence of peeling pits and debris accumulation. However, only slight delamination and debris adhesion were observed in the wear scars of the Cr coating. The Cd/Ti coating had the widest wear scars, with larger peeling pits and deeper furrows. The local enlarged image reveals an increased proportion of peeling pits, along with black coloration and an obvious layering. The wear of the Cd/Ti coating was mainly oxidation and abrasive wear, and it was the most severe. Compared with other coating techniques, the wear resistances of the Ni and Cr coatings were markedly higher, with the Cr coating showing the maximum value. The wear mechanism of the Ni coating was mainly oxidation wear accompanied by adhesive wear, while that of the Cr coating was mainly adhesive wear.

**Figure 7:**
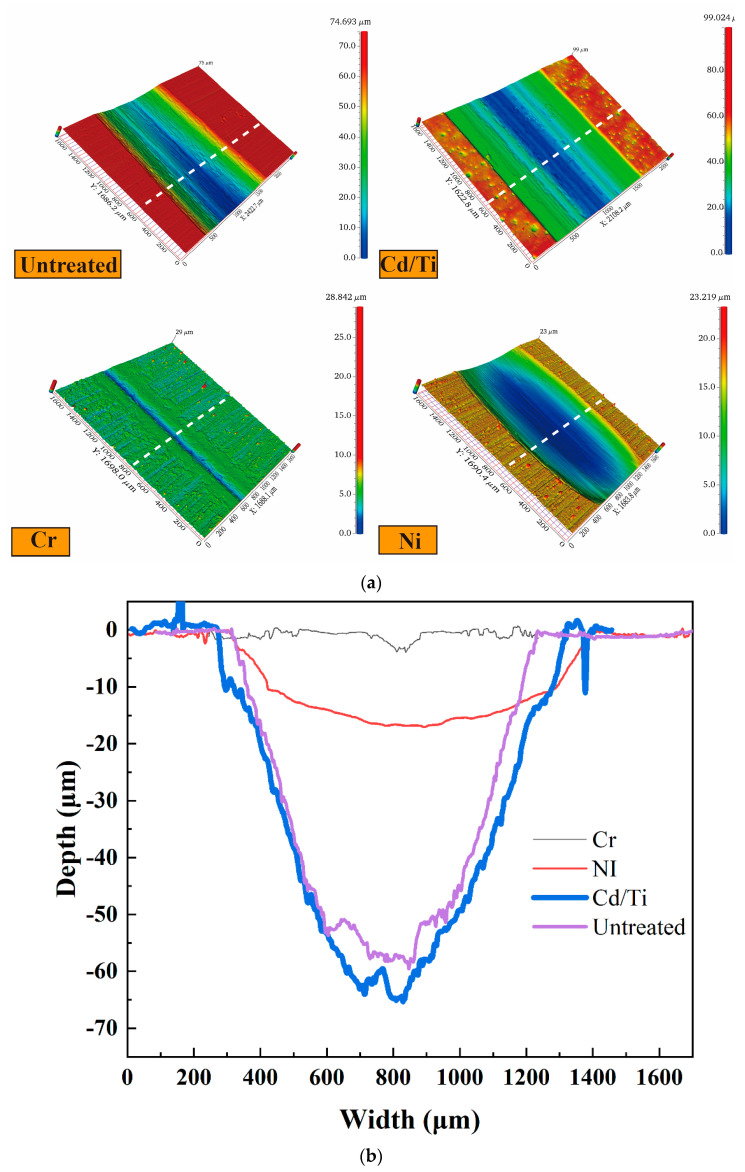
Three-dimensional morphology and cross-section profiles of different coatings. (**a**) Three-dimensional morphology of wear scars on different coatings; (**b**) Cross-section profile of wear scars on different coatings.

The three-dimensional wear morphology and cross-sectional profile of the coatings, shown in Figure 7, gave an indication of the width of the wear morphology of each coating. The difference in the maximum width in the wear morphology of the untreated samples and the Cd/Ti and Ni coatings was not substantial. Meanwhile, the width of the wear morphology of Cr coating was the smallest. Figure 7b shows the cross-sectional profiles of the wear scars on the coatings. The Cd/Ti coating had the maximum wear depth and the greatest wear degree. The wear depth of the Cr and Ni coatings was low, with the Cr coating having the minimum wear depth and the least wear degree.

**Figure 8:**
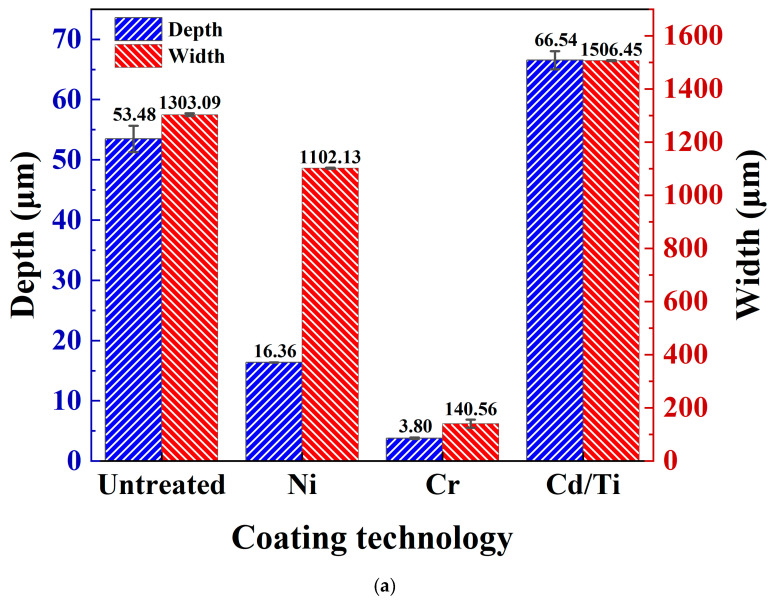

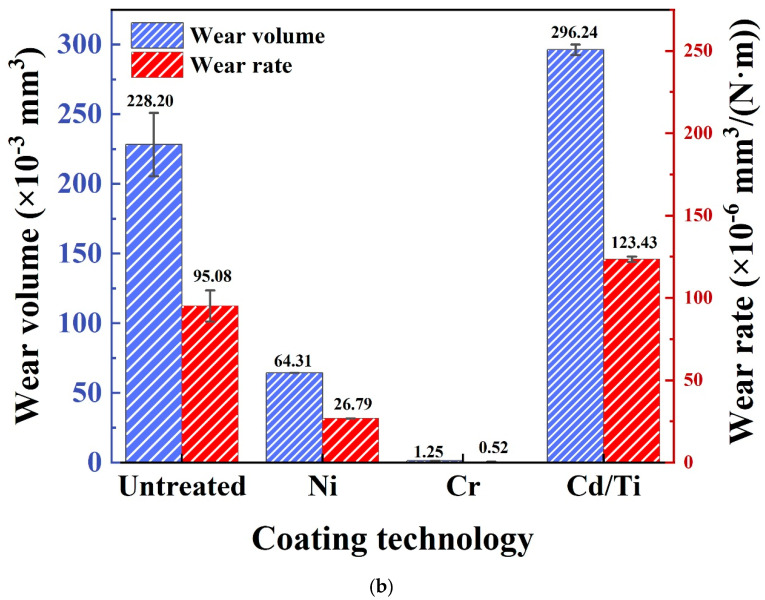
Analysis of friction and wear data. (**a**) Depth and width of wear scars; (**b**) Wear volume and wear rate.

The quantitative statistical analysis of the width and depth of the cross-section profile is shown in Figure 8a. The width and depth of the wear scars on the Cr coating decreased to about one-tenth of those of the untreated sample. The depth of the wear scars on the Ni coating decreased to about one-third of that of the untreated sample, but their widths did not decrease significantly. Compared with those of the untreated samples, the depth and width of the wear scars on the Cd/Ti coating grew to a certain extent, with an increase of about 10 μm in the depth of the wear scars.

The analysis of the wear volume and wear rate of the coatings is shown in Figure 8b. The wear volume of the Cr coating was the minimum, nearly 200 times lower than that of the untreated sample. This was in agreement with the results obtained by Zhao et al., in which chromium coating greatly improved the wear resistance of 30CrMnSiNi2A [7]. The wear rate of the coating followed the same pattern, reducing to an order of magnitude of 10^−7^, indicating good wear resistance. The wear volume and wear rate of the Ni coating decreased by nearly three times. On the other hand, the wear rate and wear volume of the Cd/Ti coating increased as compared with those of the untreated samples, with a wear rate of 10^−4^ orders of magnitude, indicating a poor wear resistance.

### 3.3. Wear Behavior

Based on the above analysis, the Cr coating had the maximum effect on improving the wear resistance of the substrate. In order to explore its wear performance under enhanced working conditions, further experimentation on the Cr coating under different stress and cycle conditions was conducted.

**Figure 9:**
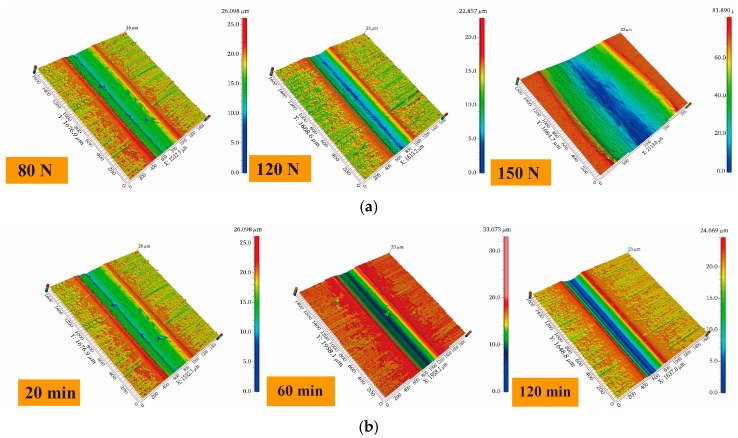
Three-dimensional wear morphology under different working conditions. (**a**) Variation of loads for a wear time of 20 min; (**b**) Variation of wear time for a load of 80 N.

The three-dimensional morphology of wear under different loads and cycle conditions is shown in Figure 9. From the three-dimensional morphology at different loads in Figure 9a, it can be clearly seen that as the load increased, the width of the wear scars gradually increased. When the load increased from 120 N to 150 N, the width of the wear scars showed a marked increase. It can be approximated that the Cr coating failed when the load was 150 N. Figure 9b shows the three-dimensional profile of wear when the wear times were increased to 60 min and 120 min under a normal load of 80 N. Here, with increasing time, the width of the wear scars did not grow significantly, but the depth of the wear scar did show growth.

**Figure 10:**
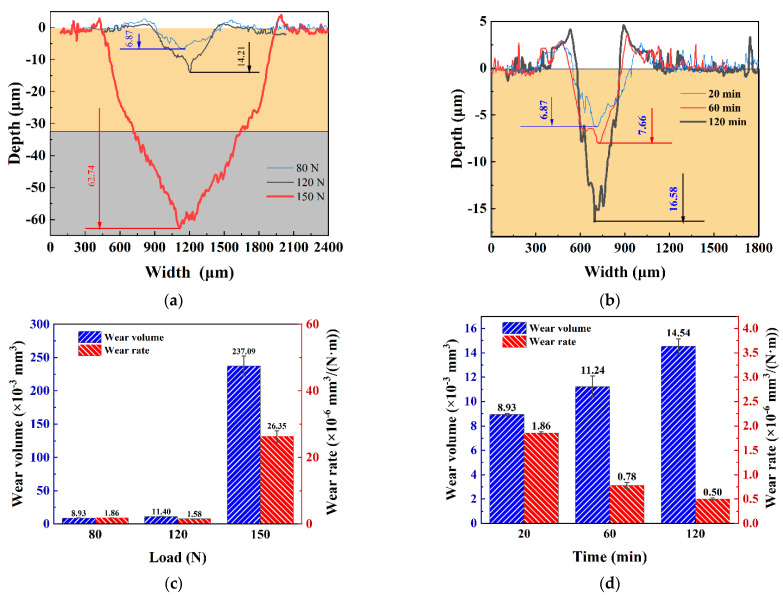
Quantitative analysis of wear scar profile. (**a**) Profile under varying load conditions for 20 min; (**b**) Profile under varying loading time conditions of 80 N; (**c**) Wear volume and wear rate under varying loads in 20 min; (**d**) Wear volume and wear rate under varying time of 80 N.

Figure 10 shows the quantitative analysis of the wear of the Cr coating under different working conditions. From the cross-sectional profiles in Figure 10a, it can be seen that when the load increased from 120 N to 150 N, the wear depth increased from 14 μm to 61.7 μm. The thickness of the Cr coating here was 33 μm. Therefore, when the Cr coating failed, the wear depth increased sharply, greatly weakening the wear resistance of the substrate. Meanwhile, the increase in the wear depth was relatively small when the load escalated from 80 N to 120 N, with an increase of only about 7.5 μm in the wear scar depth. From Figure 10c, it can be observed that the wear volume and wear rate under different loads gradually increased with rising load. The wear rate showed a trend of an initial fall and subsequent rise, similar to the wear rate of the Cr coating at 40 N in Section 3.2. As the load increased, the wear rate had three stages: (1) increase, (2) decrease, and (3) increase. This indicated that when the Cr coating did not fail, the wear rate had a maximum and a minimum point. However, when the coating did fail, its wear rate sharply increased. From Figure 10b, it can be observed that at a load of 80 N, the wear depth gradually grew with an increase in the wear time. However, it was relatively small, only about 1 μm, for an escalation from 20 min to 60 min. When the wear time went up from 60 to 120 min, there was a considerable increase in the wear depth, but the coating still did not fail. Figure 10d shows that as the wear time increased, the wear volume gradually increased, while the wear rate gradually reduced. This was because considerable debris accumulated on the wear scars, providing a lubrication effect, indicating that the impact of wear time on its wear volume was smaller than that of a normal load.

**Figure 11:**
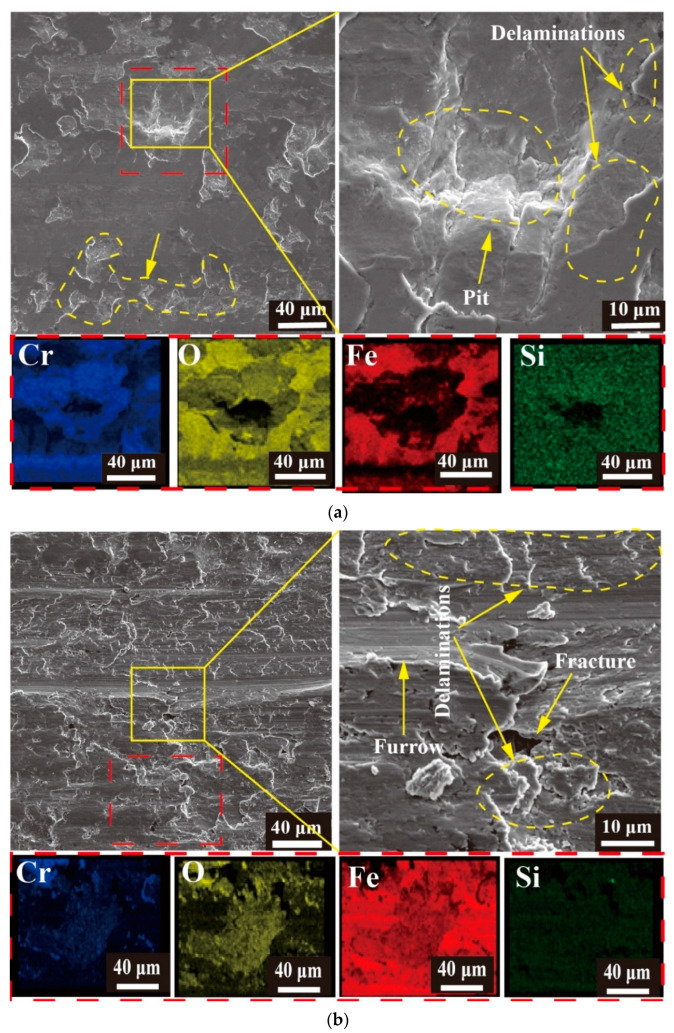

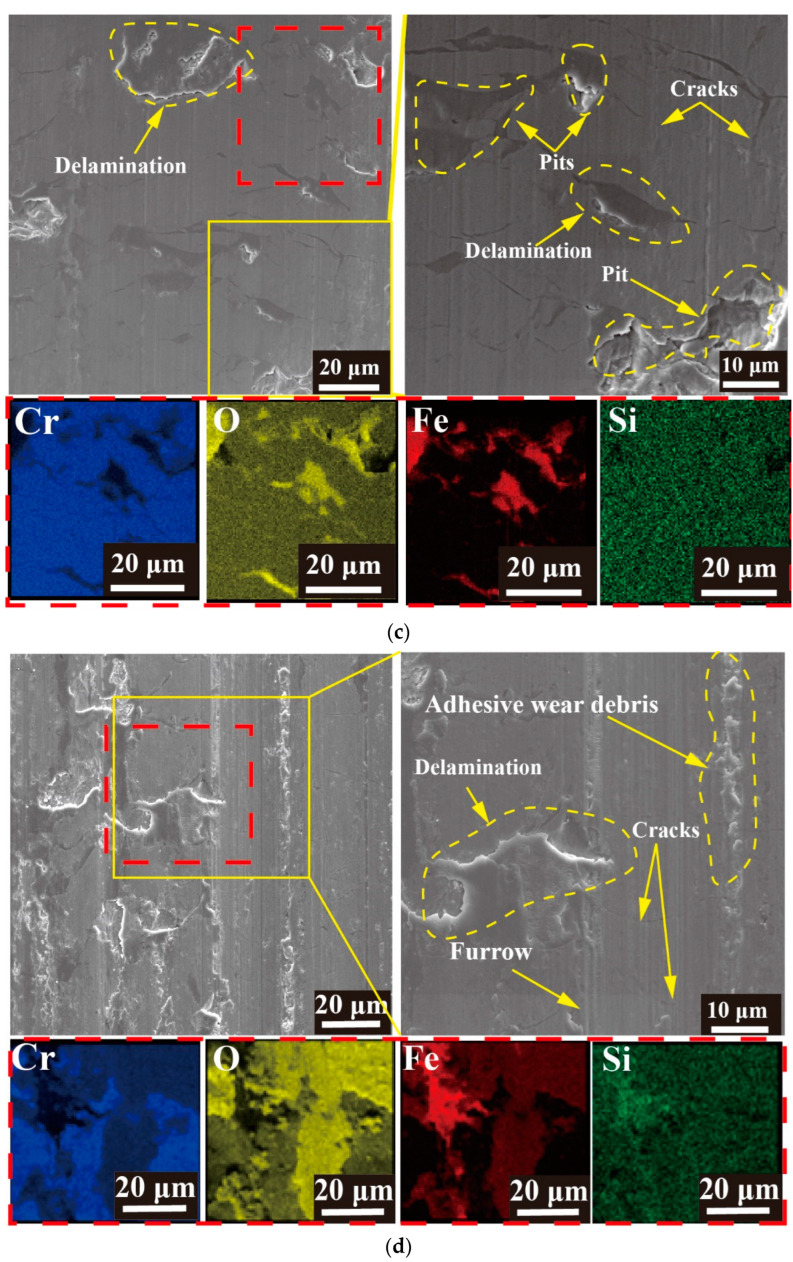
Microanalysis of wear under different working conditions. (**a**) Morphology of wear scar after 20 min of work at a load of 80 N; (**b**) Morphology of wear scar after 20 min of work at a load of 150 N; (**c**) Morphology of wear scar after 60 min of work at a load of 80 N; (**d**) Morphology of wear scar after 120 min of work at a load of 80 N.

The microstructure and elemental analyses of wear under different working conditions are shown in Figure 11. For a wear time of 20 min and a normal load of 80 N (Figure 11a), the Cr coating showed delamination and peeling pits in the wear scars [34], and there was adhesion in the wear scars. The elemental analysis shows that Cr was mainly present in the exfoliation pit, while iron oxide and silicon oxide were located around the stripped pit. Silicon and iron were elements on the grinding ball and were relatively soft. The transfer of materials also occurred from the grinding ball to the sample, resulting in severe adhesive and oxidation wear [35,36]. This was in agreement with the results of Guo et al., who investigated the effects of different temperatures on the friction and wear properties of chrome-plated layers on gun steel surfaces. The article states that the main wear mechanism at room temperature is adhesive wear, accompanied by a small amount of oxidative wear. And as the temperature increases, the degree of oxidative wear becomes greater [37].

The element analysis at a normal load of 150 N and a wear time of 20 min is shown in Figure 11b. The microanalysis indicated that the wear was more severe, and intense plastic deformation occurred in the wear scars [38,39]. Fractures and deep furrows were visible in the wear scars. From the cross-sectional profile, the coating was seen to have failed, thus destroying the substrate. Here the proportion of O in the wear scars was relatively low, and the wear scars were covered by Fe. However, the Si content in the wear scars was extremely low, as was the degree of adhesive wear. The abrasive wear was the main wear mechanism, accompanied by a small amount of oxidation wear.

For a wear time of 60 min and a normal load of 80 N (Figure 11c), large pits and delamination phenomena were observed in the wear scars, with more obvious cracks. The elemental analysis revealed that the protrusion area in the wear scar was primarily composed of Fe, Fe oxide, and Si oxide attached to the wear scar due to adhesive wear. The bottom layer was composed of Cr and Cr oxide due to oxidative wear. Under this working condition, the wear mechanism was mainly oxidation and adhesive wear.

The microstructure for a wear time of 120 min and a normal load of 80 N (Figure 11d) presented a marked accumulation of strip-shaped debris in the wear scars, with shallow cracks and furrows, as well as delamination. However, compared with that at 60 min, the surface of the wear scars was smoother because the debris accumulated in the wear scars at 60 min. Due to the friction and normal force during the friction process, the debris was compacted into the wear scars, flattening the surface of the wear scars and forming a friction layer [40]. The cracks were covered by the debris and became shallower with no pits. The elemental analysis showed that there were oxides of Fe and Cr in the wear scars, with Fe oxide aggregation at the protrusions where adhesive wear was more severe. Oxidative wear chiefly occurred at the chromium oxide aggregation.

**Figure 12:**
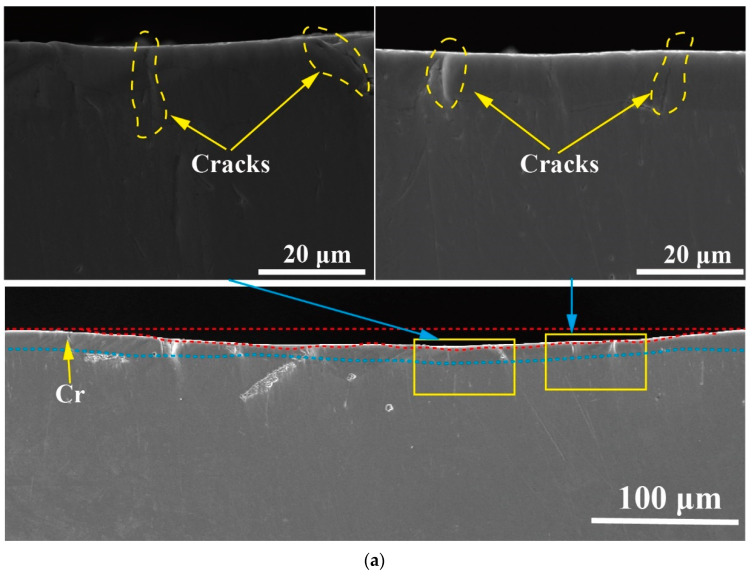

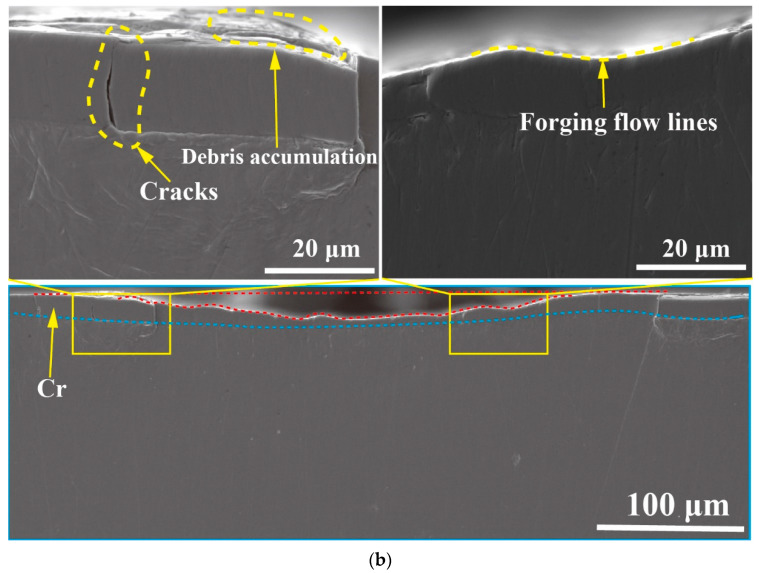
Cross-section morphology of wear at different times. (**a**) Cross-sectional morphology of wear scar after 60 min of work at a load of 80 N; (**b**) Cross-sectional morphology of wear scar after 120 min of work at a load of 80 N.

These results pointed to a lack of significant difference in wear mechanisms at a load of 80 N load for two wear times of 60 and 120 min. To further explore the differences between these two working conditions, the wear scars were cut perpendicular to the sliding direction, and their cross-sectional morphology was examined. The profile morphology of the wear scars is shown in Figure 12. Overall, the concave area and covering of the wear scars was conspicuous. The concave area was significantly deeper for the condition of 80 N for 120 min.

For the operating conditions of 80 N for 60 min (Figure 12a), many shallow cracks were observed in the cross-section, but they did not exist in the middle. They were restricted to the sides of the wear scar. The edges of the wear scar were clear of any features. This was consistent with the relatively flat morphology of the wear scar.

Figure 12b shows the cross-section of the wear scar obtained under the condition of 80 N load for 120 min. Here long cracks were present at the edge of the wear scar, which is similar to the observation in Figure 12a but more obvious. This was because during sliding, small displacements perpendicular to the sliding direction occurred due to debris buildup and vibration. This in turn caused the tangential forces at the edges to be greater than in other areas, generating long cracks at the ends [41]. Under the load of 80 N for 120 min, the unevenness of the cross-section of the wear marks was enhanced. This was due to the heat generated by friction, which reduced the hardness of the material and made it prone to deformation. On the other hand, due to the repeated squeezing effect of normal force, the material formed a structure on the surface, which was referred to as “forging flow lines” by Yu et al. [42]. As the wear time increased, the “forging flow lines” wear mode became increasingly severe.

**Figure 13:**
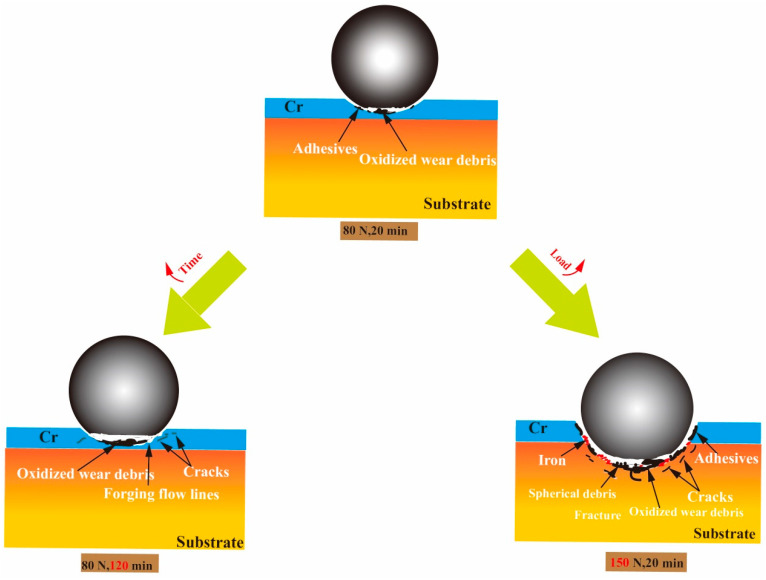
Schematic of wear mechanism.

Figure 13 is a schematic of the wear mechanism under different loads and cycle times. When the load was 80 N and the time was 20 min, the wear mechanisms were mainly adhesive and oxidation, but both were relatively mild. When the load was kept at 150 N for a time of 20 min, the Cr coating had already failed and was in the later stage of wear, with cracks and fractures appearing on the coating when the grinding ball and substrate were worn. Numerous furrows and adhesive protrusions were observed in the wear scars. Here the wear mechanisms were mainly abrasive and adhesive. When the wear time increased from 20 min to 120 min, the wear mechanism did not differ substantially. However, a small number of shallow cracks appeared in the wear scar, and the protrusion area was more obvious than that for the wear time of 20 min. Moreover, the frictional layer could be observed on the surface of the wear scars, formed by the compaction of oxidized wear materials on the surface of the wear scars.

Airplanes tend to be in more complex environments, for example, rainy environments and low- and high-temperature environments. The study of the wear resistance of each coating in different service environments is missing in this paper, and subsequent exploration can be directed to this aspect of in-depth research, making the whole study more comprehensive.

## 4. Conclusions

In this paper, the effects of different coatings (Cr, Mn, and Cd/Ti) on the wear resistance of 30CrMnSiNi2A alloy steel were investigated, and the surface chromium-plated specimens were further explored to investigate the different wear characteristics. The obtained results are given below:

1. In this paper, through the friction and wear study of different coated specimens, it was found that the chromium coating had the best wear resistance; however, the surface cadmium-plated titanium specimen had the worst wear resistance.
2. The wear performances of the coatings were corroborated by the corresponding changes in hardness and surface roughness in the coatings. The hardness of the Cr and Ni increased by three times compared with the 30CrMnSiNi2A substrate, and the wear resistance of the Cr and Ni samples to the substrate was significantly improved. After surface coating with Cd/Ti, the hardness decreased to about a third of that of the Cr/Ni coating.
3. When the load was low, the Cr coating did not fail. Here the wear was mainly oxidation and adhesive. As the time increased, small cracks appeared in the wear scars. However, the amount of wear of the coating was relatively small. This may be due to its high hardness. If only the wear time was increased without increasing the load, the Cr was not worn out easily. On the other hand, if the load was increased to 120 N and the time was kept constant, the wear depth was 14.21 μm. Here the wear mechanism was oxidation and adhesive. At a load of 150 N, the Cr coating failed completely and was devastating for the substrate. Here the wear mechanisms were abrasive and adhesive.
4. When the load was 80 N, as the wear time increased, more heat was produced by the wear. This softened the material, making the specimen more susceptible to plastic deformation. The wear cross-section of such a specimen showed “forging flow lines”.

## Figures and Tables

**Table 1:** Coating parameters for the surface conditions under study.

Parameter	Value			
Surface condition	Untreated	Chrome coating	Nickel coating	Cadmium–titanium coating
Specimen name	Untreated	Cr	Ni	Cd/Ti
Solution	N/A	CrO_3_·H_2_SO_4_ (ρ = 1.84 g/cm^3^) 2 g/L, Cr^3+^ 5 g/L	NiSO_4_·7H_2_O 25 g/L, NaH_2_PO_2_·H_2_O 15 g/L, CH_3_COONa 12 g/L, sodium citrate 12 g/L	CdCl_2_·2.5H_2_O 20 g/L, TiOCl_2_ 4 g/L, [CH_2_N(CH_2_COOH)_2_]_2_ 30 g/L, NH_4_Cl 100 g/L, NH_4_COOCH_3_ 30 g/L, (N(CH_2_COOH)_3_) 120 g/L
Anode material	N/A	Lead ladder plates containing 96% lead	Nickel sheet	Cadmium sheet
Electric current	N/A	50–60 A/dm^2^		2–3 A/dm^2^
Temperature	N/A	53–57 °C	87–92 °C	15–35 °C
Deposition rate	N/A	40–60 μm/h	12–15 μm/h	31 μm/h
Time	N/A	20 min	30 min	20 min

**Table 2:** Parameters for the friction and wear test.

Variable	Grinding Ball	Load (N)	Time (min)	Displacement Amplitude (mm)	Temperature
Coating	GCr15	40	20	5	RT
Load	GCr15	80, 120, 150	20	5	RT
Wear time	GCr15	80	20, 60, 120	5	RT

## Data Availability

The data present in this study are available upon request from the corresponding author.
